# Sustainability of ruminant livestock production in Ireland

**DOI:** 10.1093/af/vfab037

**Published:** 2021-09-06

**Authors:** Frank O′Mara, Karl G Richards, Laurence Shalloo, Trevor Donnellan, John A Finn, Gary Lanigan

**Affiliations:** 1 Teagasc, Carlow, Ireland; 2 Environment, Soils and Land Use Research Department, Teagasc, Wexford, Ireland; 3 Livestock Systems Research Department, Teagasc, Fermoy, Ireland; 4 Agricultural Economics and Farm Surveys Department, Teagasc, Athenry, Ireland

**Keywords:** biodiversity, grass-based, greenhouse gases, ruminants, sustainability

**Implications**

- Ruminant livestock production in Ireland is mainly grass based, which confers some environmental advantages in terms of manure recycling, soil organic carbon content, feed self-sufficiency (including protein), amount of human-edible food in the diet, greenhouse gas emissions per kilogram of product, and landscape diversity.
- Environmental policy and targets relating to greenhouse gas and ammonia emissions, water quality, and biodiversity will require widespread adoption of new technologies and changes to the production system.
- Research is needed to identify new mitigation technologies and changes to farming practice that can allow environmental targets to be reached without compromising food production.

## Introduction

The relationship between agriculture and the environment is significant. Agriculture is the main economic use of land globally. The rising global population, particularly over the last century, has required a vast increase in agricultural activity. While it was necessary to focus on producing more food in the last century, there is increasing recognition that for the future, the focus within agriculture needs to be increasing production in a sustainable way that reflects the economic, social, and environmental goals of society. In this context, Ireland provides an interesting case study of a country where agriculture is dominated by relatively extensive grass-based ruminant production which confers some positive environmental characteristics, but yet agriculture in Ireland is facing many challenges to decrease environmental impact and meet environmental regulations, while at the same time remaining economically sustainable.

This paper provides some background on Irish agriculture. It sets out the rationale that ruminant production systems based largely on a grazed grass diet are more sustainable food production systems than systems that import a large proportion of the animals’ diet onto the farm in the form of concentrate feed, when taken in the context of land quality and the opportunities for alternative land use. This paper also accepts that there are many aspects that can improve through solutions that are developed and need to be applied as well as solutions currently not yet discovered. The paper then details the international and domestic environmental policy pressures on Irish agriculture, with a particular focus on greenhouse gas (**GHG**) emissions given the intense focus on this issue nationally and internationally. Next, the research taking place to improve environmental performance, particularly GHG emissions, is described, as are the metrics developed to measure progress. The final section provides concluding remarks.

## Irish Agriculture in Context

Agriculture in Ireland is dominated by livestock, with 88% of gross agricultural output coming from livestock products (CSO, 2020). In turn, livestock production in Ireland is dominated by ruminants, which comprise 87.5% of the value of livestock output. Dairy, beef, and sheep farms make up the bulk of the farms in Ireland, which are mostly family run and owned. There are also important pig and poultry industries, with most of their output coming from a relatively small number of units of considerable scale. Crop production is a less significant land use than in much of the European Union (**EU**) because of climate, farm structures, and land suitability, with a report completed in the early 1980s stating that approximately 31% of Irish agricultural land was suitable for crop production (Gardiner et al., 1980).

**Figure 1.**
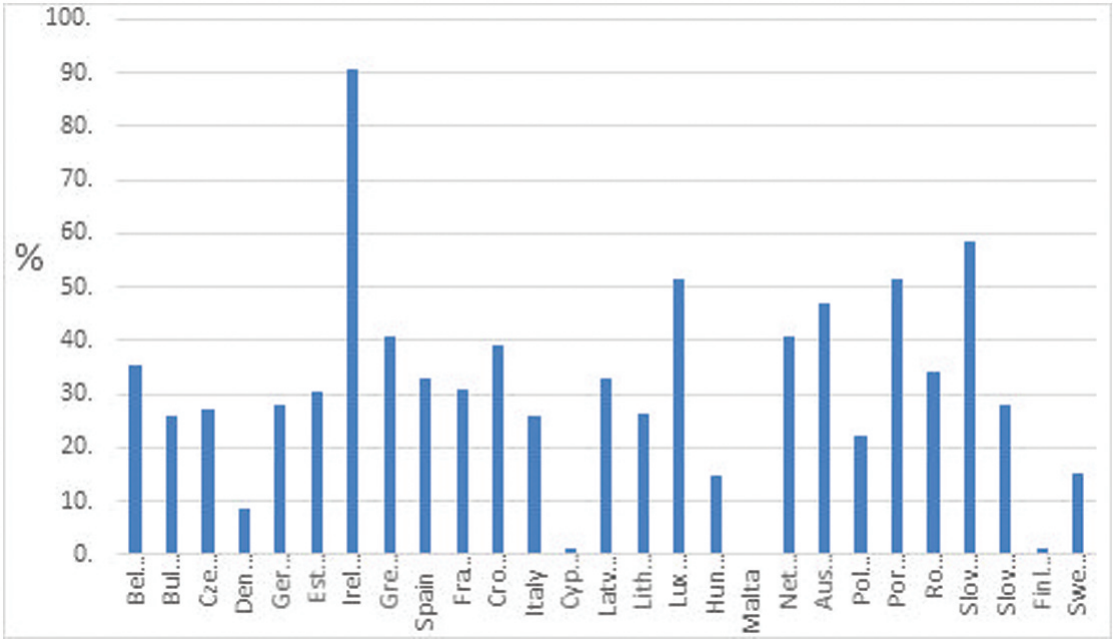
Share of utilized agriculture land under grassland in EU states in 2016 (Source: Eurostat, 2021).

The ruminant livestock industries that dominate Irish agriculture are predominantly grass-based systems. Data from Eurostat show that Ireland had by far the highest percentage of utilized agricultural area under grassland at 90.4% in 2016 with Slovenia being the next highest at 58.4% (Figure 1).

Irish grass-based systems of milk and meat production rely on the conversion of human inedible forage into highly nutritious and digestible human-edible products. An important feature of Irish systems has been the selection of animals to suit forage-based systems, which has been particularly successful within dairying. Dairy cows and bulls are selected based on an economic breeding index (**EBI**), which places as much weighting on fertility traits as productivity traits, resulting in better animal fertility and higher animal performance. Calving date is used to synchronize feed demand and feed supply in the form of available grazed grass. The focus of the system is to turn cows out to grass as soon as possible post calving to maximize the proportion of the diet coming from grazed grass. Both stocking rate and concentrate supplementation are used to strategically and tactically manage the interface between feed supply and feed demand. As a result, livestock in ruminant enterprises are predominantly fed on grazed or conserved forage, mainly grass. O′Brien et al. (2018) reported that the average diet of dairy cows (based on a representative set of farms in the Teagasc National Farm Survey (**NFS**) for the years 2013–2015) was 81.8% forage, with concentrates constituting just 18.2% of the annual feed budget on a dry matter basis. Of the 81.8% forage, 60.2% was grazed pasture, 19.8% was grass silage, and 1.8% was alternative forages. Equivalent data on dietary composition for beef cow herds or sheep flocks are not available from the NFS, but information from Teagasc Roadmaps (Teagasc, 2020) for beef systems and Earle et al. (2017) for sheep systems indicates similar or higher levels of forage in the diet.

The NFS 2019 Sustainability Report (Buckley and Donnellan, 2020) indicates that the average dairy farm stocking rate is just under 2.1 livestock units (**LU**)/Ha, whereas cattle rearing and cattle finishing farms have a stocking rate of about 1.4 LU/Ha on average. A dairy cow is one grazing livestock unit and other animals are given equivalent values (see Donnellan et al., 2020, for further details). For environmental reasons, a maximum stocking rate of 170 kg of organic nitrogen excretion per ha is allowed, unless a farm has a derogation from the EU Nitrates Directive (EC, 1991), which allows the stocking rate to increase to 250 kg of organic N excretion per ha. A typical Irish dairy cow excretes 91 kg of organic N per year. Therefore, the stocking rate limit for dairy farms with a derogation is 2.75LU/Ha.

The environmental consequences of the grass-based system are discussed in the next section.

## Environmental Characteristics of Irish Livestock Production Systems

The high reliance on grazed and conserved forage and generally moderate to low stocking rates means that ruminant livestock production in Ireland has strong underlying environmental advantages in terms of manure recycling, soil organic carbon content, feed self-sufficiency (including protein), amount of human-edible food in the diet, GHG emissions per kilogram of product (GHG intensity, defined as kg of CO_2_ equivalent per kg of milk or meat), and landscape diversity. The grazing system, which almost always incorporates the production of silage for winter fodder on the same farm, means that feed production and livestock are very closely integrated on the same farm with very good circularity and recycling of animal manures. The high feed self-sufficiency resulting from the low feed imports onto the farm (e.g., 18.2% concentrates in dairy cow diets as outlined above) means that N and P surpluses are relatively low and below the EU average (see later discussion). As grassland soils hold higher soil organic carbon stocks than arable soils and given the predominance of grassland in Ireland, Irish soils have high contents of organic matter and store large quantities of carbon, with evidence suggesting that they continue to sequester carbon over time. Simo et al. (2019) reported soil organic C stock in Irish mineral soils to be 499 Mt, equivalent to 1,832 Mt CO_2_e, an amount equivalent to approximately 30 times Ireland’s total annual GHG emissions.

Another key feature of sustainability is limiting competition for land for feed and food production. There is very little human-edible food in the diet of Irish ruminants, and they largely convert inedible cellulose-based feeds to high-quality human foods. The diets are predominantly forage based, and the concentrate component of the diet is mainly constituted of byproducts of oilseeds and grains, with typically just 25 percent whole grains (O′Brien et al., 2018). In a recent analysis, Hennessy et al. (2021) reported that the edible protein conversion ratio of Irish dairy systems (including beef production of the progeny not used for replacements) was 0.21, that is, 0.21 kg of human-edible protein was consumed for every 1 kg produced. The corresponding figure for beef cow systems was 0.43, again indicating more human-edible protein is produced than consumed. In an analysis completed by Laisse et al. (2018), the corresponding figure for total mixed ration-based system in France was 1.0. Hennessy et al. (2021) further analyzed the land use ratio (**LUR**; Van Zanten et al., 2016), which considers the fact that some of the land used for feed production could have been used for food production. The dairy (and dairy beef) system had a LUR of 0.51, indicating that it is very efficient at converting human-inedible food into digestible protein. The corresponding figure for beef cow systems was 1.78, suggesting that converting some of the land used for beef cow production to human-edible food production would improve this metric. However, there are economic, farm system, farm structure, and demographic barriers to make this type of change difficult at an individual farm level without significant policy interventions. Any change in system needs to be evaluated from the perspective of all indicators including for example potential loss of soil carbon and biodiversity which could arise from the conversion of a grazing livestock system to food crop production.

The reliance on grazed grass and the selection of animals to suit forage-based systems are positive for the emissions intensity of milk production. In a study that evaluated the impact of stocking rate and supplementary feed on GHG emissions, it was shown that increasing stocking rate was associated with reduced emissions per unit of the product, while increasing concentrate supplementation had the opposite effect (O′Brien et al., 2010). In general, the focus of the system is on minimizing the use of supplementary feed and maximizing the use of grazed grass. This is done by ensuring that the grazing season length is as long as possible with previous research showing that increasing the length of the grazing season is associated with reduced GHG emissions per unit of product (Lovett et al., 2008). Breeding for higher EBI in dairy cows has been shown to reduce GHG emissions per unit of product by 1% for a €10 increase in EBI (Lahart et al., 2021) while at the same time increasing profitability (O′Sullivan et al., 2020). The GHG emissions from Irish livestock systems are discussed further below.

The mixture of pasture and grazing livestock with an extensive network of hedges and stone walls forming field boundaries creates an attractive and diverse landscape. The Irish national average enclosed field size is only 2.5 ha (Zimmermann, 2018). Green et al. (2019) reported that there is 690,000 km of hedgerows, of all types, in Ireland, with an average width of 2.7 m, and that they occupy 2.6% of the land area. Excluding commonage, there is approximately 4.5 million ha of utilized agricultural land in Ireland in 2016 (CSO, 2018). Thus, there is an average length of hedgerow of c. 150 m per ha, and the figure for grassland farms is likely to be higher than the average as field size tends to be larger on tillage farms.

Nevertheless, despite these positive sustainability features, Irish livestock production has significant challenges to meet environmental policy restrictions, regulations, and targets in the areas of GHG and ammonia emissions, water quality, and biodiversity.

## Policy and Targets for Environmental Performance of Irish Livestock Production Systems

The EU sets national GHG reduction targets for member states via the European Union Climate and Energy Framework and subsequent Effort Sharing Proposals (COM/2016/482). In its Climate Action Plan 2019, the Irish government set a target for a 10% to 15% reduction in agricultural GHG emissions by 2030 relative to projected 2030 emissions. This is a challenging target given that the Irish bovine herd increased by 8.6% from 2010 to 2019 (a 41% increase in the dairy cow herd facilitated by the relaxation and eventual removal of the EU milk quota system in 2015 and a concomitant but smaller reduction in beef cow numbers). Lanigan et al. (2018) projected that emissions from agriculture would rise over the period to 2030 in the absence of mitigation due to increased agricultural activity (mainly increased dairy cows and their progeny used for beef production), but with adoption of a range of mitigation measures, a 15% reduction in GHG emissions was possible. Some of the efficiency-related measures would require a restriction on agricultural production to make them fully effective. Additional GHG offsetting by land use carbon sequestration and fossil fuel displacement were also outlined by Lanigan et al. (2018).

The European Green Deal (EC, 2019) has set a higher level of ambition for GHG reduction, and, currently in Ireland, a new Climate Action and Low Carbon Development Bill (Government of Ireland, 2021) is expected to be enacted in 2021. This will sign into law a 51% reduction in GHG emissions by 2030, and while sectoral targets are not yet defined, it is highly likely that agriculture will have to deliver greater than the 10% to 15% reductions in emissions under this revised legislation than in the previous Climate Action Plan 2019 (Government of Ireland, 2019). The new Bill will also set out that the distinct characteristics of biogenic methane are to be considered.

Ammonia emissions are regulated in the EU and Ireland by the National Emission Ceilings Directive (**NECD**) (EC, 2016). As in most EU Member States, ammonia emissions in Ireland are predominantly from agriculture at 98% of the total (Duffy et al., 2015). The current target under the NECD is for a 0.5% decrease compared with 2005 levels. However it is likely that a proposed target of a 5% reduction by 2030 compared with 2005 levels will be agreed. Buckley et al. (2020) estimated the maximum technical abatement potential would result in this emissions target just about being met, should the adoption rates of the various mitigation measures be achieved. Data on ammonia emissions in different ruminant livestock systems are presented in Table 1. Many of the strategies to mitigate ammonia emissions are also strategies to reduce nitrous oxide emissions, and ammonia is not discussed further in this paper.

**Table 1.** Average Irish GHG and ammonia emissions intensity of livestock production in Ireland in 2019 from the Teagasc NFS using the IPCC^*a*^ and LCA^*a*^ methods (Buckley and Donnellan, 2020)

	GHG			Ammonia	
	Unit	Calculation method		Unit	
		IPCC	LCA		
Milk	Kg CO_2_ eq/kg FPCM^*b*^	0.777	1.14	Kg NH_3_/kg FPCM	0.0057
Beef	Kg CO_2_ eq/kg LW output^*c*^	12.28		Kg NH_3_/kg LW output	0.0598
Sheep	Kg CO_2_ eq/kg LW output	8.5		Kg NH_3_/kg LW output	0.017

^*a*^See explanations for the methodologies in the text.

^*b*^FPCM: fat- and protein-corrected milk, corrected to 4.0% fat and 3.3% protein.

^*c*^Liveweight (LW) output: the kilogram of liveweight gained per beef animal or sheep.

Water quality is regulated in the EU and Ireland by the Water Framework Directive (**WFD**; EC, 2000), which requires at least “good” water quality in all EU water bodies (rivers, lakes, groundwater, and transitional coastal waters). In Ireland, this must be achieved by 2027. The EU Nitrates Directive (EC, 1991) has been implemented in Ireland since 2007 and regulates agricultural practices related to the WFD, such as stocking rate, fertilizer use, manure storage requirement, and timing of manure and fertilizer application. From a water quality perspective, Ireland’s statistics are better than most other EU countries with 53% of Irish surface waters at good or high status compared with 44% in the EU and 92% of groundwater being good compared with 80% in the EU (Wall et al., 2020). But there are challenges due to the decline in high status waters and increasing eutrophication. In its latest assessment of water quality, the Irish Environmental Protection Agency (**EPA**) reported that a trend over the last decade toward improvement in water quality had stopped, and there was a slight decline from 55% of surface water bodies having either good or high ecological health in the last assessment period of 2010–2015 to 52.8% in the most recent assessment (EPA, 2020b). The report highlighted the challenges in the south and east of Ireland where water quality was declining and agricultural pressures were higher. The report also noted that agriculture was a significant pressure on Ireland’s aquatic environment. Because of the relationship of nutrient losses with water quality as well as climate change, ammonia emissions, and biodiversity in water bodies, the recently published EU Farm to Fork Strategy (EC, 2020), a part of the European Green Deal (EC, 2019), calls for a 50% reduction in nutrient (especially N and P) losses and a 20% reduction in fertilizer use. The performance of livestock systems in relation to water quality and nutrient loss is discussed below.

In Ireland, the basic designation for biodiversity is the natural heritage area, for habitats or for species of plants and animals whose habitat requires protections. The EU Habitats Directive prescribes a list of habitats and species considered to be important at European scale that must be protected within special areas of conservation. Under the EU Birds Directive (2009/147/EC), Ireland is required to designate Special Protection areas for the protection of: listed rare and vulnerable species, regularly occurring migratory species, and wetlands especially those of international importance. Outside of protected areas, relatively little is known about the state of biodiversity. In their recent “State of the Environment” report, the Irish EPA assessed Ireland’s biodiversity as being “very poor,” “Deteriorating trends dominate, especially for protected habitats...,” and “Largely not on track to meet policy objectives” (EPA, 2020b). Recognizing the current global threat to biodiversity, the EU has committed to the Convention on Biological Diversity’s aim to “ensure that by 2050 all of the world’s ecosystems are restored, resilient, and adequately protected”. In the meantime, the EU “aims to ensure that Europe’s biodiversity will be on the path to recovery by 2030.” Some high-level aims of the EU Farm to Fork and Biodiversity strategies include: at least 10% of the agricultural area is under high-diversity landscape features; 30% of the land will be protected and connected through ecological corridors; and the decline in pollinators is reversed.

## Livestock Production and GHG Emissions

### Agricultural emissions

Agriculture accounted for 35.3% of Ireland’s GHG emissions in 2019 (EPA, 2021). These emissions that are reported to the United Nations Framework Convention on Climate Change (**UNFCCC**) are calculated using methodologies provided in the 2006 Intergovernmental Panel on Climate Change (**IPCC**) Guidelines for National Greenhouse Gas Inventories (IPCC, 2006). Ireland’s agricultural emissions as a percentage of national emissions for 2018 are high by international standards, with the EU-27 average and the United Kingdom being 10.1% and 8.2%, respectively (EEA, 2021) and the USA being 9.3% (EPA, 2020a). New Zealand is one of the few developed countries to have a higher share of emissions coming from agriculture than Ireland. In New Zealand, agricultural emissions in 2018 accounted for 48% of total emissions (Ministry for the Environment, 2020). Both Ireland and New Zealand share the characteristics of having a low population density, little heavy industry, and a high share of agriculture in their economic activity. For example, there are 1.4 bovines for every person in Ireland compared with 0.14 per person in the United Kingdom.

**Figure 2.**
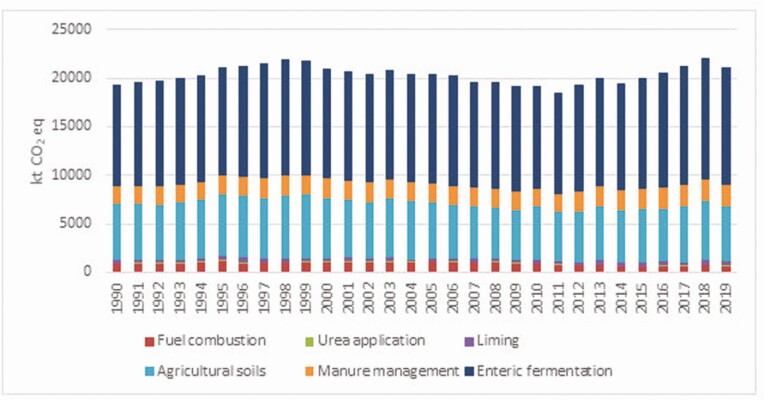
Total GHG emissions from Irish Agriculture by source, 1990–2019 (Source: EPA, 2021).

Irish agricultural emissions peaked in 1998, and declined for most of the period to 2011, but have been rising since (Figure 2). These trends closely match the size of the cattle herd (peak in 1998 at 7.59 million head), which is the main driver of emissions, with nitrogen fertilizer use (peak in 1999 at 443,000 tonnes) being the other significant driver. Relative to 1998, by 2011, the cattle herd had dropped over 1 million head to 6.43 million and nitrogen fertilizer use fell to 296,000 tonnes in the same year, a decrease of close to 150,000 tonnes on the 1998 level.

**Figure 3.**
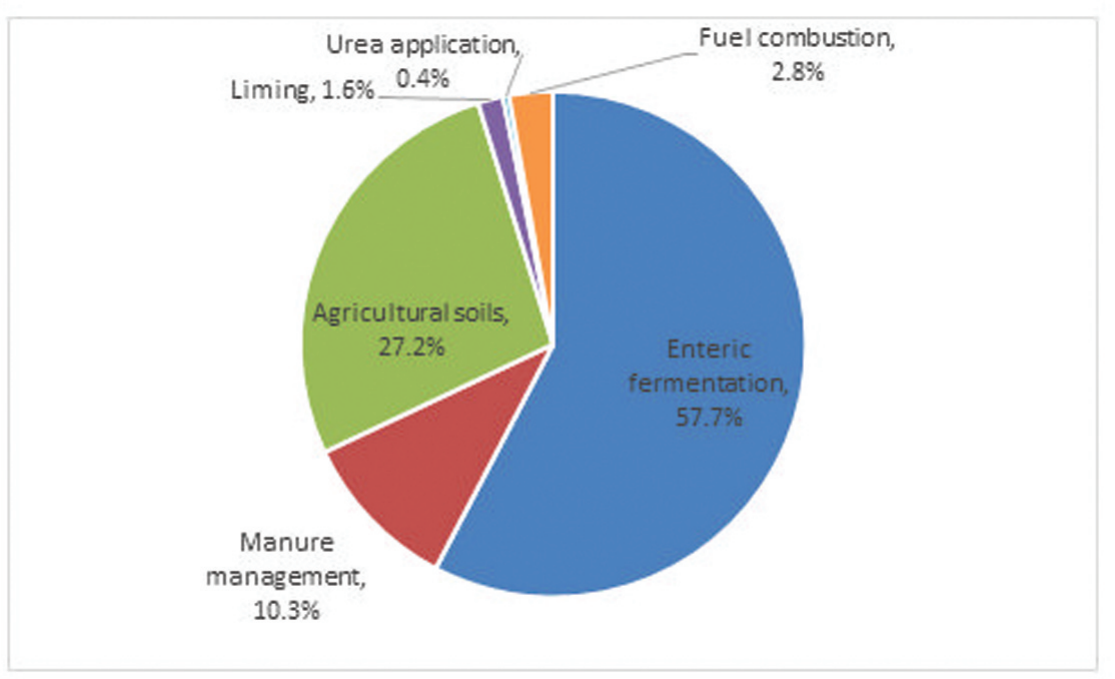
Breakdown of agricultural GHG emissions by source for 2019 (Source: EPA, 2021).

Livestock are the main source of agricultural GHG emissions in Ireland, accounting for about 90% of the total, reflecting their high share of agricultural activity in Ireland. Using the Food and Agricultural Policy Research Institute Ireland model, Donnellan (2021, personal communication) has calculated that above 80% of agricultural GHG emissions arise from bovines and other livestock make up about 8% of the total. The sources of GHG are shown in Figure 3 with methane from enteric fermentation accounting for above 57.9% of agricultural emissions in 2018 which reflects the large cattle herd (EPA, 2020c).

### Emissions intensity of livestock production in Ireland

As shown above, livestock dominate Irish agricultural GHG emissions by virtue of the dominance of the livestock sector in Irish agriculture but this does not give any indication of the efficiency of the Irish livestock sector in terms of emissions per kilogram of milk or meat produced. The GHG emissions intensity of Irish milk production was reported by Leip et al. (2010) to be the joint lowest in the EU, at just under 1 kg CO_2_ eq per kg milk within the system boundaries of that study. This is based on milk production data from 2005 which is quite dated now, but it does represent a detailed study by the Joint Research Centre of the European Commission and carried out with a consistent methodology across all member states. For beef, the study reported that Ireland had the fifth lowest emissions intensity.

Detailed metrics based on nationally representative data are available to illustrate how emissions intensity is evolving in recent years. In the 2019 Teagasc NFS Sustainability Report, Buckley and Donnellan (2020) calculated dairy GHG emissions using a life cycle analysis (**LCA**) approach, accounting for all emissions including inputs brought onto the farm (e.g. feed, fertilizer, and energy) that are used in the production system, regardless of whether they are sourced domestically or internationally. Using this approach, they reported GHG emissions to be 1.14 kg CO_2_ eq per kg of fat and protein corrected milk (**FPCM**) in 2019 for the average farmer (Table 1) with the weighted average milk pool closer to 1.1 kg CO_2_ eq per kg of FPCM. Interestingly, similar to previous analysis (O′Brien et al., 2015), they showed farms with a better economic performance had a lower emissions intensity per unit of output. They also reported the LCA-derived emissions over time on a 3-yr rolling average basis from 2013–2015 to 2017–2019 (Table 2). The decline reflects improvements in production efficiency, such as the output of FPCM/cow and adoption of mitigation technologies. For instance, protected urea, which has lower nitrous oxide emissions than calcium ammonia nitrate fertilizer, is used on 13% of dairy farms in 2019 (Buckley and Donnellan, 2020). Recent research has established Tier 2 emissions factors for nitrogen fertilizers and animal manure (Harty et al., 2016; Krol et al., 2016), and these are due to be incorporated into the models calculating emissions in the NFS dataset. These new emission factors will reduce the footprint derived from the NFS dataset by between 0.1 and 0.14 kg CO_2_ eq per kg of FPCM, to a figure of approximately 1.0 kg CO_2_ eq per kg of FPCM.

**Table 2.** Average Irish GHG emission intensity per kilogram of product over time on dairy, cattle, and sheep farms from the Teagasc NFS using the IPCC^*a*^ and LCA^*a*^ methods (Buckley and Donnellan, 2020)

	Unit	Method	2014	2019
Dairy	Kg CO_2_ eq/kg FPCM^*b*^	LCA	1.23	1.14
Cattle	Kg CO_2_ eq/kg LW output^*c*^	IPCC	13.7	12.3
Sheep	Kg CO_2_ eq/kg LW output	IPCC	10.2	8.9

^*a*^See explanations for the methodologies in the text.

^*b*^FPCM: Fat- and protein-corrected milk, corrected to 4.0% fat and 3.3% protein.

^*c*^Liveweight (LW) output: the kilogram of liveweight gained per beef animal or sheep.

Buckley and Donnellan (2020) report that, in 2019, the average cattle and sheep farms emitted 12.28 and 8.5kg CO_2_ eq per kg of beef or sheep produced, respectively. This is calculated using the IPCC national inventory methodology for agricultural emissions in national inventory reports and does not account for emissions outside the country associated with the production of imported feedstuffs and fertilizers (Table 1). It does include emissions associated with energy use on farms. As with dairy cows, cattle and sheep farms with better economic performance had a lower emissions intensity per unit of output. The emissions per kilogram of liveweight produced are observed to decline over time (Table 2) due to better efficiency and some adoption of mitigation technologies such as protected urea (which has lower nitrous oxide emissions than the other nitrogen fertilizers such as calcium ammonium nitrate), although adoption of protected urea was lower on cattle farms than dairy farms and very limited on sheep farms.

### Technologies to reduce GHG emissions

Research in Ireland and internationally has been used to develop a Marginal Abatement Cost Curve for agricultural GHG emissions (Lanigan et al., 2018, Figure 4). The individual measures and their mean mitigation potential over the years 2021–2030 are outlined in Table 3. Continued gains in efficiency by, for instance, improvement in dairy cow genetics, as measured by the EBI, have the potential to reduce emissions. However, if cow numbers continue to increase, the impact of increases in EBI on absolute GHG emissions is more limited. There are also a range of technological measures that will reduce emissions directly, such as changing fertilizer type (use of urea-based fertilizers in place of calcium ammonium nitrate), reduction in the overall chemical nitrogen use, reduced crude protein content in animal feedstuffs, and spreading of slurry using low emissions technology.

**Figure 4.**
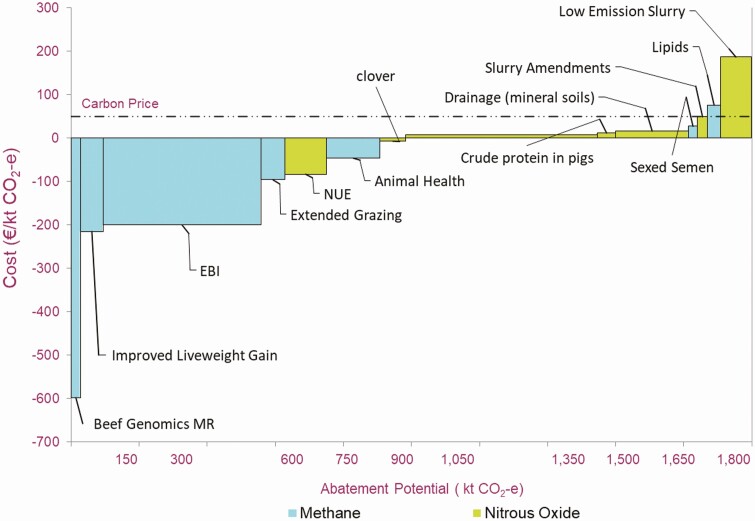
The marginal abatement cost curve for Irish agriculture for 2021–2030 (methane and nitrous oxide abatement). Values are based on linear uptake of measures between the years 2021 and 2030 and represent the mean yearly abatement over this period. Measures principally impacting methane are indicated in green and N_2_O in blue. The dashed line indicates Carbon cost of €50 per tonne CO_2_ (taken from Lanigan et al., 2018). Beef MRI, Beef Maternal Replacement Index, an index of beef cow genetic merit; Dairy EBI, an index of dairy cow genetic merit.

**Table 3.** Measures to reduce agricultural GHG emissions and their mean mitigation potential over the years 2021–2030

Measure	Mean annual abatement Mt CO_2_e
Improved beef maternal traits (CH_4_)	0.03
Beef genetics: liveweight gain (CH_4_)	0.06
Dairy genetics: improve EBI (CH_4_)	0.43
Extended grazing (CH_4_)	0.07
Nitrogen-use efficiency (N_2_O)	0.1
Improved animal health (CH_4_)	0.1
Sexed semen (CH_4_)	0.02
Inclusion of clover in pasture (N_2_O)	0.07
Change fertilizer type (N_2_O)	0.52
Reduce crude protein in pig diets (N_2_O)	0.05
Draining wet mineral soils (N_2_O)	0.2
Slurry amendments (CH_4_)	0.03
Adding fatty acids to dairy diets (CH_4_)	0.03
Low emissions slurry spreading (N_2_O)	0.12
Total	1.83

Source: Lanigan et al. (2018).

In addition to these currently available technologies, there is an active research program in Ireland, which is linked internationally that is searching for additional ways to reduce emissions of methane, nitrous oxide, carbon dioxide and enhancing soil carbon sequestration. These technologies and farm practices include feed additives, dietary oils, halides and seaweed, livestock breeding, animal lifetime efficiency, optimization of soil pH and nutrient levels, changes in fertilizer type and amount used, manure acidification, multi-species swards, increasing grass quality including clover, as well as rewetting agricultural peat soils, and enhancing soil fertility and the soil microbiome.

### Summary on livestock and GHG emissions

Agriculture and livestock make up a large share of GHG emissions in Ireland, but this masks the fact that this is a sector which by international comparison has low emissions per unit of output, thus making a sustainable contribution to global food security. Indeed, studies by the EU Joint Research Centre on the impact of 2030 targets on EU agriculture found that implementation of a *pro-rata* reduction across the component parts of the non-emissions traded sector sector resulted both in reduced agricultural production in most member states and *a net increase* in global agricultural emissions as production moves to less emissions efficient countries (Fellmann et al., 2018). It concluded that net imported emissions should be considered when setting national mitigation targets. Nevertheless, the high (and rising) absolute emissions result in domestic pressure to reduce emissions, and the future growth or indeed reduction in the size of the national herd is being actively debated. Therefore, it is important for the sector to find practical and economically feasible ways to reduce emissions. Some technologies to reduce emissions are available and other research is underway. There is a lot of interest in carbon sequestration in soils, hedges, and woodlands/forests on farms to offset emissions. Lanigan et al. (2018) have identified several practices that could increase carbon sequestration and a new soil carbon observatory with an associated research program is being established (see https://agri-i.ie/portfolio-items/c-sequestration/ for details). The high share of emissions from biogenic methane means that the treatment of biogenic methane by the IPCC and UNFCCC is very important for Ireland especially as strategies are developed to achieve climate neutrality by 2050. A clear understanding and strategy around the most appropriate metrics and methodologies will have a very significant impact on the prioritization of research investment in the appropriate areas needed.

Finally, conversion of some land to biofuel/biomass production could help offset emissions in other parts of the economy, while potentially taking land away from ruminants. It is important to note if this land is food producing (tillage), it more than likely will impact the LURs, while if the land is currently in pasture the release of carbon involved in cropping will be significant.

## Livestock Production and Water Quality

### N and P balance and efficiency of use in Irish agriculture

**Figure 5.**
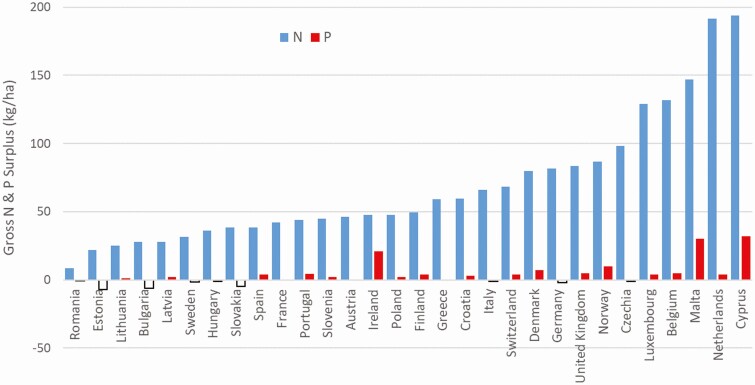
Nitrogen (N) and phosphorus (P) surplus for EU countries in 2015 (Source: Eurostat, 2019).

Agricultural systems in different countries can be compared for their pressure on water resources by examining gross N and P balances, which indicate the potential nutrient surplus in kilogram N and P per ha per year. The latest comparison for the year 2015 (Eurostat, 2019) indicates that Ireland has a national N and P surplus of 42 kg N and 5 kg P/ha, respectively, which is below average for member states (Figure 5). Nutrient balances at a national level will be influenced by the mix of enterprises among other factors, and Quemada et al. (2020) recently reported that arable systems had a lower N surplus than livestock or mixed systems. As Irish agriculture is dominated by livestock production as outlined above, the low nutrient balances are indicative of the comparatively extensive nature of Irish livestock systems.

Based on the representative set of farms in the NFS, Buckley and Donnellan (2020) reported that N and P balances were significantly higher on dairy farms compared with beef or sheep farms. On a 3-yr rolling average basis (2017–2019), N balances were 184, 67, and 58 kg/ha/yr on dairy beef and sheep farms, respectively, and the corresponding figures for P balance were 13.3, 6.1, and 7.1 kg/ha/yr. (Table 4). N surpluses have increased since the 2012–2014 period, especially for dairy and sheep farms. This is at least partly related to a severe drought in 2018, which resulted in large increases in purchased feed on most of the farms to counteract the deficit in grass grown. P balances have also increased which is related to regulatory changes in 2014 that allowed additional P to be applied where soils had sub-optimal soil P levels.

**Table 4.** Average nitrogen and phosphorus balance^*a*^ and efficiency^*b*^ of use on Irish dairy, cattle, and sheep farms from the Teagasc NFS (Buckley and Donnellan, 2020)

	N balance/ha		P balance/ha	
Subsector	2012–2014	2017–2019	2012–2014	2017–2019
Dairy	170	184	9.2	13.3
Cattle	63	67	5.0	6.1
Sheep	51	58	5.0	7.1
	Nutrient use efficiency (%)			
Dairy	21.4	23.4	60.6	53.3
Cattle	21.3	22.4	66.4	66.8
Sheep	26.8	28.5	64.6	57.6

^*a*^Balance is the N or P inputs minus N or P offtakes in animal products.

^*b*^Efficiency is N or P offtakes/N or P inputs, expressed as a percentage.

Nutrient use efficiency has been improving on Irish dairy farms (Buckley and Donnellan, 2020). The average dairy farm had an N and P use efficiency (NUE and PUE) of 23.4% and 53.3%, respectively (Table 4), and the top-performing farmers had higher nutrient use efficiency (Buckley and Donnellan, 2020). Dutch dairy farms had an average NUE and PUE of 23% to 26% and 30% to 34%, respectively (Oenema and Oenema, 2021). Estimates of nutrient use efficiency typically do not take account of efficiency associated with imported feed or manure exported from farms (Quemada et al., 2020) or nutrients that are immobilized or sequestered in soil. Irish dairy farms operate a zero P balance where inputs match outputs, but generally soil P levels are low which results in P build up in soil. This reduces the P available for grass growth and this is not reflected in the PUE. There is scope to further improve NUE and PUE on Irish farms through improving soil pH (liming) and through low emission slurry storage and spreading to increase manure nitrogen availability (Lanigan et al., 2018).

### Improving water quality

The largest threat to water quality in Ireland is from eutrophication due to excessive nutrients in water. More recently, sediment was highlighted as the most pervasive stressor on water quality and more important than nutrient concentration (Davis et al. 2018) Nutrient loss to water follows the source–pathway–receptor model where you need to have a source of nutrients, a pathway for nutrients to move in the landscape, and a sensitive receptor or receiving water. Improving water quality requires site-specific advice and interventions by farmers. The source and pathways for nutrients and sediment differ and targeting critical source areas for each nutrient is needed. Nutrient hotspots have been identified in Ireland (Mockler et al., 2017) and these are being targeted by the Local Authority Waters Programme (Lawpro) to identify nutrient sources and then the Teagasc Agricultural Sustainability Support Service (ASSAP) are providing farmers with free one-to-one advice on what measures are needed to improve the water quality in their area. Advisors are working collaboratively with farmers, local authorities, and the agri-food industry to improve water quality (Micha et al., 2018). The range of measures that are being recommended to farmers or subject to ongoing research is summarized in Table 5 below. A key focus in all of the strategies and especially in the context of climate change is the movement away from a prescriptive structure around nutrient use to a focus on precision in the use of all farm nutrients from a timing to a location perspective.

**Table 5.** Source and pathway measures to reduce nitrogen and phosphorus loss to water and improve water quality

	Nitrogen	Phosphorus
Source measures	Nutrient management planning	Nutrient management planning
	Reduce nitrogen fertilizer through optimizing soil fertility	Optimize soil fertility—only apply P where required
	Reduce fertilizer through optimizing manure management	Optimize spatial distribution of manure to optimize inorganic P fertilizer use
	Precision nutrient management—avoiding application at high-risk times	Precision nutrient management—avoiding application at high-risk times
	Precision fertilizer management—avoid application to high-risk areas (riparian buffers)	Precision fertilizer management—avoid application to high-risk areas (riparian buffers)
	Incorporate legumes in swards to reduce fertilizer use	Soil-specific P fertilizer advice to optimize P availability for crop
	Use nitrification inhibitors	Reduce P loadings
	Reduce nitrogen loading from fertilizer and manure	
Pathway measures	Reduce connectivity between farmyards and receiving waters	Reduce connectivity between farmyards and receiving waters
	Reduce cattle access to surface waters	Reduce cattle access to surface waters
	Controlled drainage	Intercept critical source area breakthrough and delivery points
	Riparian buffer zones	In ditch remediation using vegetation, sediment traps, and reactive media
	Break connectivity between dry ditches, roadways, underpasses, and waters	Break connectivity between dry ditches, roadways, underpasses, and waters
	Constructed wetlands	Constructed wetlands
	Denitrifying bioreactors	Permeable reactive barriers
		Stream bank side stabilization

## Livestock Production and Biodiversity

**Figure 6.**
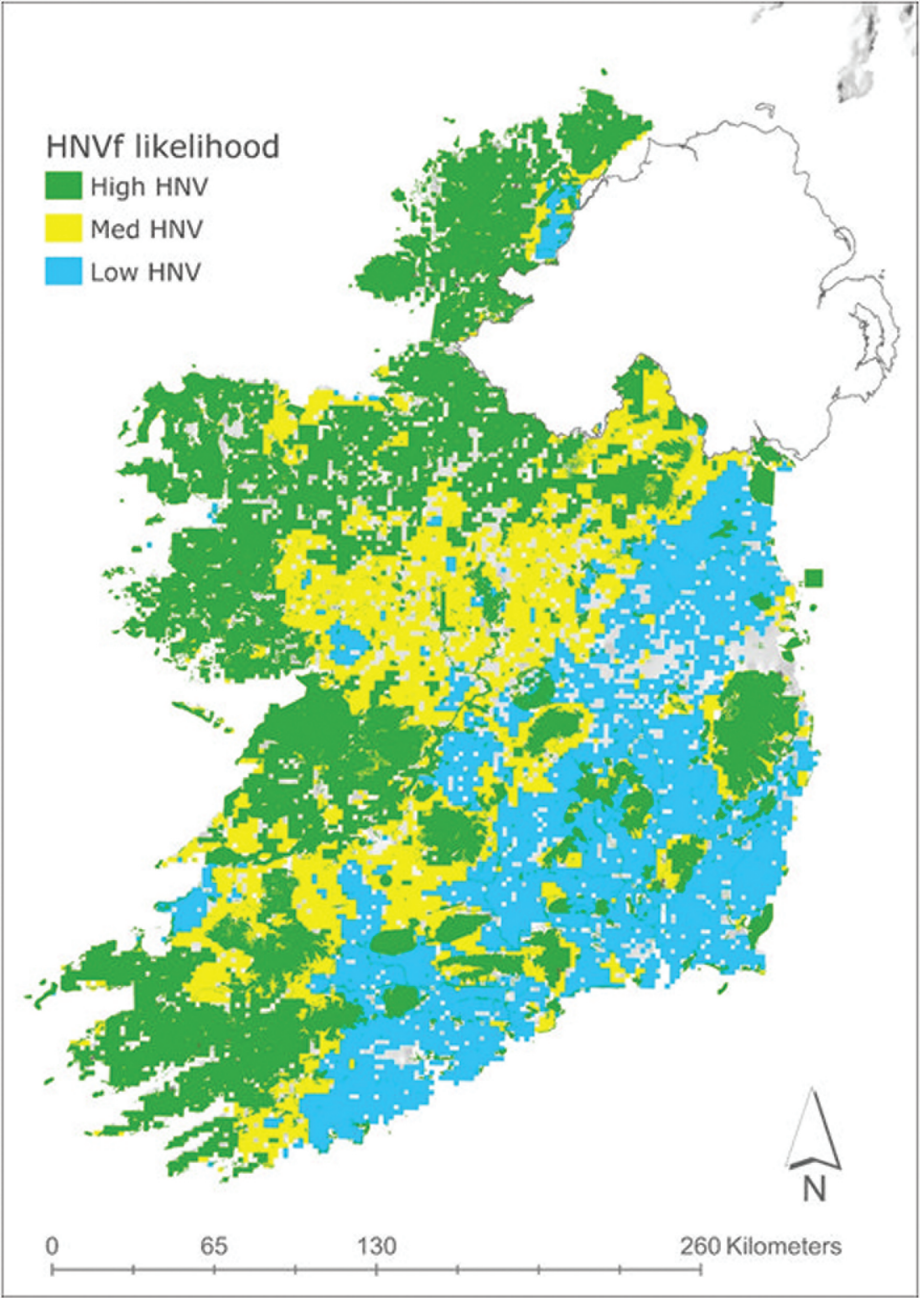
Predicted distribution of high nature value farmland (HNVf) in the Republic of Ireland (adapted from Matin et al., 2020).

Over generations, traditional livestock systems have shaped much of Ireland’s farmland biodiversity. High nature value (**HNV**) farming systems are widely distributed across the western seaboard, uplands, and some other areas and are strongly associated with more extensive livestock systems (Figure 6) (Matin et al., 2020). HNV farming systems occupy about a third of farmland (Matin et al., 2020) and are associated with the majority of the farming system comprising semi-natural habitat. More intensively managed livestock systems are associated with lower levels and diversity of semi-natural habitats. Looking at a gradient of farming intensity from extensive to intermediate to intensive, the Farm-Ecos project in Ireland showed that the area of semi-natural habitat declined from 42% to 15.6% to 6.1%, respectively (Rotchés-Ribalta et al., 2021). Surveys of mostly grassland farms in Ireland reported average semi-natural habitat areas of 13% to 15% (Sheridan et al., 2011, 2017; Sullivan et al., 2011). In an Irish study of more intensively managed farms (*n* = 119), the wildlife habitat area across three separate farming enterprises found a median farm habitat area of 5% for tillage, 6% for intensive beef, and 6.55% for intensive dairy (Larkin et al., 2019).

A wider biodiversity impact of livestock systems arises from the conversion of tropical rainforest for the cultivation of soy to supplement animal intake of protein. Importantly, biodiversity impacts due to off-farm land use change can be as large as those that occur on-farm (Teillard et al., 2016); the greater the reliance on off-farm feed from biodiversity hotspots, the greater the impact. In this regard, the low reliance of Irish livestock systems on concentrate feeds is a positive feature.

## Summary and Conclusions

Agriculture in Ireland is dominated by grass-based ruminant livestock production. Stocking rates are low to moderate on most farms and highest on dairy farms. Maximizing the use of grazed grass is a central feature of the systems, and emphasis is placed on breeding animals suited to grass-based systems, especially for dairying. These characteristics confer some environmental advantages in terms of good crop and livestock circularity and manure recycling, low food vs. feed competition, high soil organic matter and carbon sequestration potential, contribution to biodiversity, and low GHG intensity of milk and meat. Nevertheless, there are also significant challenges in terms of absolute GHG and ammonia emissions, water quality, and biodiversity. Milk and meat produced in Ireland have GHG emissions intensity per kilogram of milk and meat that are among the lowest in the world, but international agreements to reduce absolute emissions mean that Irish livestock systems are under pressure to reduce production to comply. Ireland has good water quality by international standards, but progress toward required standards has stalled in recent years. Biodiversity is an issue, particularly on some intensively stocked farms. So, while Irish livestock production systems have many positive fundamental attributes, continued evolution and improvements are necessary over the coming years to decrease environmental impact and meet environmental regulations. Some technologies and system adaptations have been identified that will improve performance, and the challenge for knowledge transfer systems is to achieve widespread adoption of these. Research is underway to identify new technologies and pathways for further improvements in environmental performance. It is a significant challenge for research, knowledge transfer, farmers and the agri-food industry to find and deploy these solutions at a pace, scale and location sufficient to meet the timeframe of environmental targets.

## About the Authors

**Frank P. O′Mara** is the Director of Research, Teagasc. Graduated from University College Dublin with a PhD in ruminant nutrition, he lectured and researched in UCD for 13 years before moving to Teagasc and into research management in 2006. His research interests are mainly sustainable livestock production systems.

**John A. Finn** is the Researcher in Biodiversity and Agri-Ecology, Teagasc. Graduated from University College Cork with a PhD in community ecology, he lectured and researched at UCC and the University of Reading before joining Teagasc in 2002. His research interests include multi-species grassland mixtures, results-based approaches for ecosystem services, High Nature Value farming systems, and effective actions for biodiversity restoration in intensively farmed landscapes.

**Trevor Donnellan** is the Head of Agricultural Economics and Farm Surveys at Teagasc. He is a Business and Economics graduate of University College Cork, where he also worked before joining Teagasc. His research interests include the ex-ante analysis of agricultural policy, trade policy, and environmental policy, including greenhouse gas emissions.

**Karl G. Richards** is the Head of Environment, Soils and Land Use Department, Teagasc. Graduated from Trinity College Dublin with a PhD in Environmental Geology, he worked as a consultant hydrogeologist Dames & Moore followed by scientific officer in the research division of the Environmental Protection Agency before joining Teagasc 19 years ago. His research interests include understanding and mitigating nitrogen dynamics in agricultural soils, nitrate leaching, and denitrification in croplands and grazed grasslands soils.

**Laurence Shalloo** is a Research Scientist, Animal and Grassland Research and Innovation Department, Teagasc. He is also a Systems research scientist with interests in modeling the overall sustainability of pasture-based systems of milk production as well as integration of precision-based technologies and solutions within the decision-making process at the farm level.

**Gary J. Lanigan** is a researcher in agricultural greenhouse gas emissions. Graduated from University College Dublin with a PhD in plant physiology, he previously held research/lecturing posts at Cambridge University and Trinity College Dublin. His research interests include measuring and modeling the impacts of management and climate on ecosystem carbon and nitrogen cycling.
